# Comparing the Field and Laboratory Curing Behaviour of Cold Recycled Asphalt Mixtures for Binder Courses

**DOI:** 10.3390/ma13214697

**Published:** 2020-10-22

**Authors:** Gilda Ferrotti, Andrea Grilli, Chiara Mignini, Andrea Graziani

**Affiliations:** 1 Department of Civil and Building Engineering and Architecture, Polytechnic University of Marche, 60131 Ancona, Italy; c.mignini@pm.univpm.it (C.M.); a.graziani@staff.univpm.it (A.G.); 2 Department of Economics, Science and Law, University of the Republic of San Marino, 47890 San Marino City, San Marino; andrea.grilli@unirsm.sm

**Keywords:** cold recycling, curing, indirect tensile strength, indirect tensile modulus, reclaimed asphalt, bituminous emulsion

## Abstract

The cold recycling of reclaimed asphalt (RA) for the rehabilitation of end-of-life pavements is becoming very common. Cold recycled asphalt mixtures (CRAMs) are characterised by a curing time, required to reach the material design mechanical performance. Since the laboratory simulation of the long-term field curing is not yet a standardised procedure, a CRAM was laid as binder course in a full-scale trial section that was monitored for more than two years. The comparison between field curing and oven-curing in laboratory at 40 °C was performed by carrying out indirect tensile stiffness modulus (ITSM), indirect tensile strength (ITS) and complex modulus tests, as well as measurements of the air voids content. The evolution of the ITSM as a function of the curing time (field/oven-curing) was obtained for both gyratory specimens and cores taken from the trial section at different time periods. Results showed that the material stiffness development can be accelerated with a small effect on its long-term value if oven-curing is applied a few days/weeks after compaction. A linear relationship was found between the ITS measured on the cores and their air voids content. Finally, the complex modulus tests confirmed that CRAMs provide an intermediate behaviour between asphalt concrete mixtures and cement-bound mixtures.

## 1. Introduction

In the rehabilitation of end-of-life pavements, the focus on cold recycled materials (CRMs) is strongly on the rise, allowing the reuse of reclaimed asphalt (RA) in a cost-effective and environmentally friendly way [1].

The use of different binding agents (i.e., emulsified or foamed bitumen and cement) and their dosages lead to the acquisition of different types of CRM [2], such as bitumen stabilised materials (BSMs), cold recycled asphalt mixtures (CRAMs) and cement–bitumen treated materials (CBTMs) [3,4]. The CRM mechanical behaviour is strictly linked to its composition [5], providing mixtures with properties ranging from those similar to unbound granular materials [6] to those of materials characterised by an intermediate behaviour between cement-bound mixtures [3,7,8] and asphalt concrete (AC). For this reason, several authors have characterised the mechanical behaviour of CRAM by using the complex modulus [9,10,11]. For CRAMs and CBTMs, the combined effect of bitumen and cement is very effective in terms of water sensitivity [7,8,12,13,14] and high-temperature stability [12,13,15].

The CRM composition in terms of RA content, is mainly related to the recycling technique [9,16,17]. Cold in-place recycling (CIR) is carried out directly at the jobsite and involves only the bituminous layers. Therefore, the RA aggregate content may reach 100% of the aggregate blend. Full-depth reclamation (FDR) [8,18] is also carried out at the jobsite and involves the full-depth of the pavements, including the bituminous layers and the underlying granular or cemented layers. In this case, the RA content may be reduced to about 30%. Cold central-plant recycling (CCPR) is carried out in a fixed or mobile plant, where selected RA aggregate and virgin aggregate can be combined in the optimal way. The possibility to better control the proportion of the mixture components during material production with CCPR makes this technology able to produce high-performance CRMs for binder and base courses [17].

The selection of the most appropriate CRM composition and recycling technique depends on several factors such as equipment and material availability, thickness and level of the pavement degradation, as well as economic considerations. CRM reaches its design mechanical properties in terms of strength and stiffness only after a specific curing period [19]. Depending on the CRM composition, the curing process results from a combination of several mechanisms such as emulsion breaking, moisture loss and hydration of cementitious compounds [4,7,20,21,22]. The curing in the field is a gradual process which may require some weeks or even months and is affected by several factors such as temperature, relative humidity, drainage conditions, and layer thickness [4,23]. Specifically, higher temperatures accelerate the curing [23,24], whereas the influence of the relative humidity is mainly linked to the presence of cement [25]. Indeed, a higher humidity promotes the formation of cementitious bonds, thus increasing the material stiffness.

To investigate the long-term performance of the CRMs, the curing is usually accelerated in laboratory by subjecting the specimens to oven-curing at a fixed temperature for several days. Many procedures for curing acceleration exist [26]. The most widespread consists of oven-cure CRM specimens at 40 °C for 3 days immediately after compaction, even if it does not seem to guarantee the achievement of the maximum mixture performance [27].

The main objective of this study was to compare the long-term behaviour of field-cured and laboratory-cured CRAM mixtures. To this end, a trial section was carried out by applying, as binder course, a CRAM produced in an asphalt plant (CCPR technique). Laboratory specimens were compacted immediately after CRAM production and cores were taken from the trial section at several time intervals (up to 796 days after construction). The evolution of the indirect tensile stiffness modulus (ITSM) was studied in different curing conditions: oven-curing at 40 °C, in the field and in a combination of field and oven-curing. Moreover, the relationship between indirect tensile strength (ITS) and air voids content was obtained. Finally, the rheological characterisation of the studied CRAM was performed by measuring and modelling the complex modulus of field specimens.

## 2. Experimental Program

### 2.1. Materials and Mixture

The CRAM consists of RA aggregate, virgin river sand, mineral filler, bituminous emulsion, cement and water.

The main characteristics of the RA aggregate and river sand are shown in Table 1 and Table 2, respectively. A mineral filler with high fineness (Table 3) was chosen to increase the fine content in the blend and improve the bituminous mastic consistency.

An over-stabilised bituminous emulsion, designated as C60B10 (EN 13808 [39]) and a Portland limestone cement, designated as CEM II/B-LL 32.5R (EN 197-1 [40]) were selected as co-binders. Their main characteristics are shown in Table 4 and Table 5.

**Figure 1:**
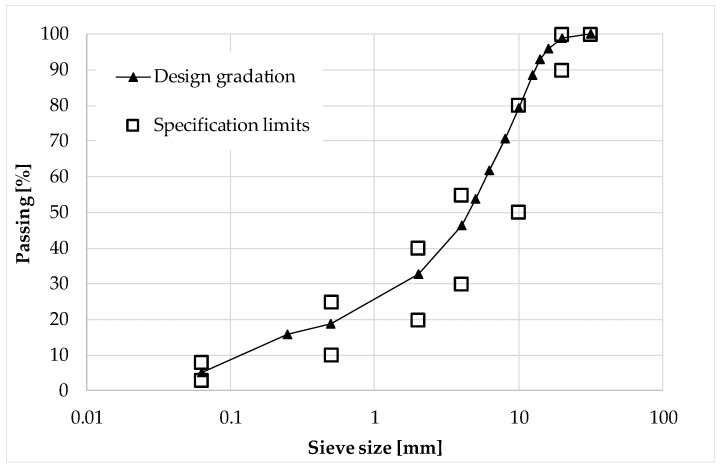
Design gradation.

The mix design of the CRAMs employed in this research was carried out in accordance with the specifications of the Republic of San Marino road agency [51]. The design aggregate blend was obtained by combining 88% of RA aggregate, 10% of river sand and 2% of filler (Table 6 and Figure 1). The design bituminous emulsion dosage, by dry aggregate mass, was 4.5% and 4.0% (2.7% and 2.4% of residual bitumen) for the mixes employed for the binder and base courses, respectively. The design cement dosage, by dry aggregate mass, was 2.0% and the total water content was 5.0% by dry aggregate mass (including both the emulsion water and prewetting water). At the design composition, the CRAM specimens compacted with a gyratory compactor at 100 gyrations had a dry density of 2.123 Mg/m^3^ (EN 12697-6 [52]) and an average indirect tensile strength (ITS) (EN 12697-23 [53]) value at 25 °C of 0.41 MPa (curing for 72 h at 40 °C).

### 2.2. Trial Section

#### 2.2.1. Mixing Plant

**Figure 2:**
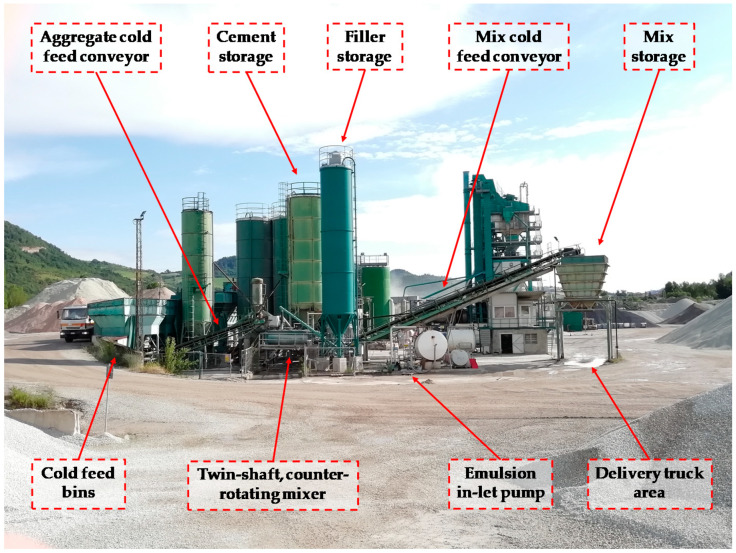
Mixing plant for cold mixture production.

To produce the CRAM, a mix plant for cement treated mixtures was modified by adding an inlet and storage system for the bituminous emulsion (Figure 2). The emulsion is directly discharged at around 55 °C into the twin-shaft counter-rotating mixer, almost simultaneously with aggregates, filler, cement and water. The mixing requires 20 s and afterwards the cold mixture is transferred into a storage bin before being discharged into waiting trucks. The capacity of the mixing plant is 200 t/h.

#### 2.2.2. Construction of the Trial Section

**Figure 3:**
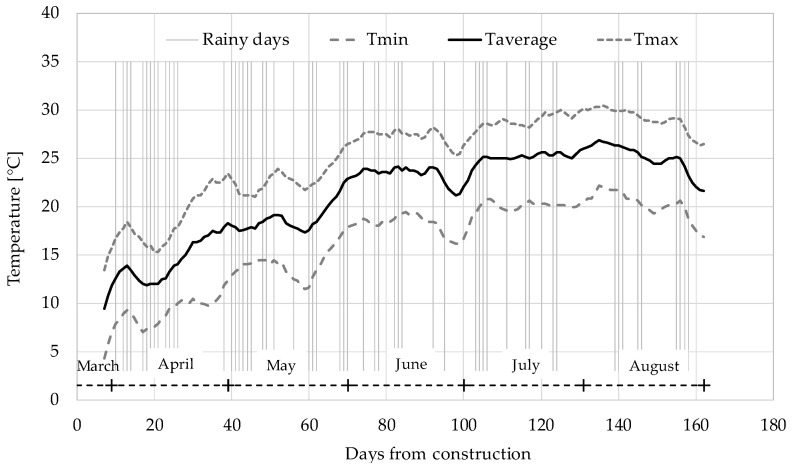
Rainy days (vertical lines) and moving average (period = 7 days) of daily temperatures near the plant.

The trial section was constructed adjacent to the mixing plant that is located in central-northern Italy, where a Mediterranean climate is present. Since the curing of CRAMs strongly depends on climatic conditions, the daily temperatures and the rainy days were collected at a meteorological station 35 km away from the mixing plant. They are shown in Figure 3, starting from the day of production and laying (24 March 2018) until about 5 months later (31 August 2018).

**Figure 4:**
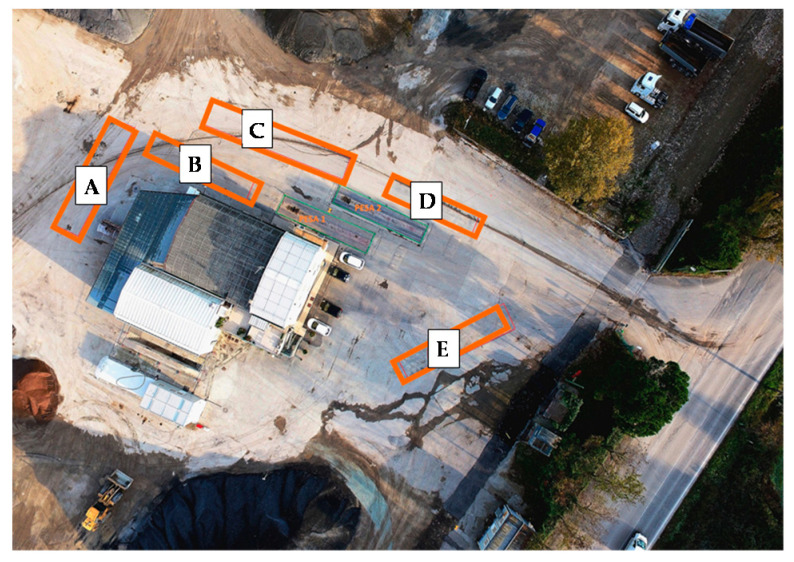
Location of the five Subsections.

**Figure 5:**
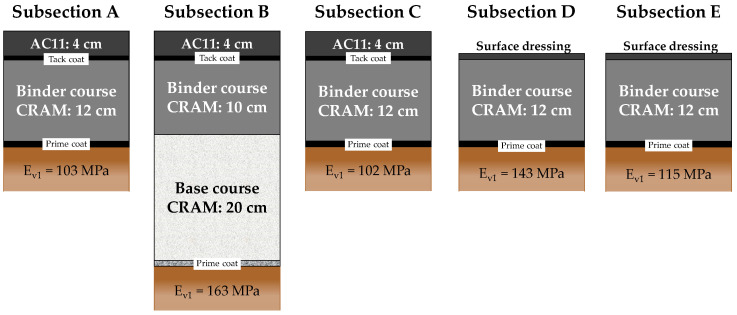
Scheme of the Subsections composition.

The trial section (Figure 4), detailly described by Grilli et al. [54], consists of five subsections (A, B, C, D and E) characterised by different layers (Figure 5) and different compaction procedures of the CRAM binder course (Table 7). Before construction, static plate load tests (DIN 18134 [55]) were performed on the subgrade of all subsections and the E_v1_ modulus results are shown in Figure 5.

Three types of structure were considered to simulate different maintenance activities provided by the Republic of San Marino road agency:

- Subsections A (17 m long and 5 m wide) and C (25 m long and 4 m wide) represent a typical intermediate maintenance work and consist in 12 cm of CRAM binder course and 4 cm of asphalt concrete wearing course with maximum aggregate size of 11 mm (AC11);
- Subsection B (25 m long and 4 m wide) represents a typical deep maintenance work and consists of 20 cm of CRAM base course, 10 cm of CRAM binder course and 4 cm of AC11 wearing course;
- Subsection D (15 m long and 4 m wide) and E (19 m long and 4 m wide) represent a typical maintenance work for rural roads and consists of 12 cm of CRAM binder course and a double-layered surface dressing.

In all subsections, a bituminous emulsion prime coat (1.00 kg/m^2^ of residual bitumen) was applied above the subgrade and a bituminous emulsion tack coat (0.60 kg/m^2^ of residual bitumen), or a surface treatment, was applied above the CRAM layer, immediately after construction.

**Figure 6:**
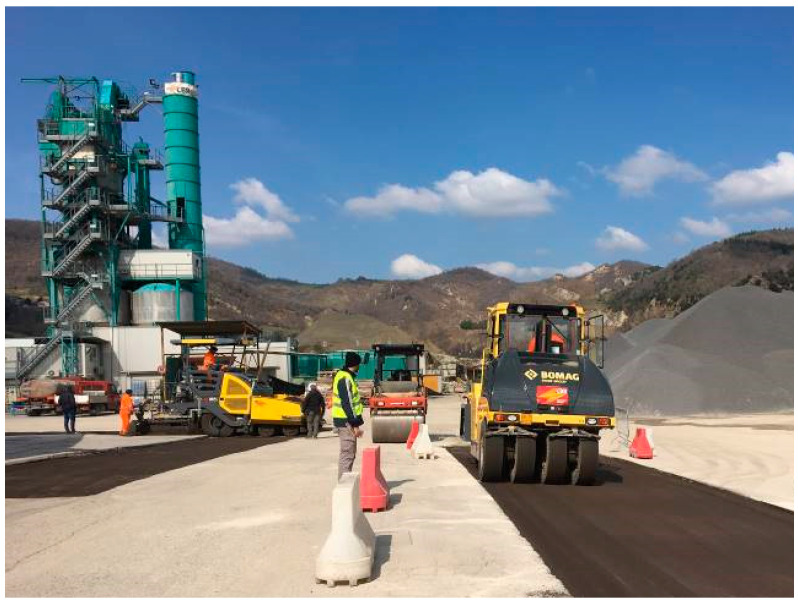
Compaction of CRAM binder course of Subsection C by using the 22-ton pneumatic-tired roller.

Several compaction procedures, in terms of roller weight (9, 12 or 22 tons), roller type (steel or pneumatic) and number of passes (back-forth movement), were considered for the CRAM binder course to take into account different construction practices (Table 7 and Figure 6). The roller covered the width of the layer in no more than four overlapping passes assuming that the overlap is not less than 20 cm.

All the Subsections were opened to traffic 4 days after construction. Traffic data were collected for Subsection B, C and D. After two years from construction, Subsection B was subjected to heavy traffic corresponding to about 270,000 cycles of 80 kN equivalent standard axle load (ESAL) whereas Subsections C and D were subjected to about 70,000 ESAL. No distresses are visible on the Subsections.

### 2.3. Experimental Program

In this study, only the CRAM applied as binder course (Figure 5) was tested. During the construction of the trial section, the loose mixture was immediately compacted with a gyratory compactor to produce four specimens with a diameter of 150 mm and a height, after 100 gyrations, of about 70 mm. After the trial section construction, 30 cores were taken from the subsections at several time intervals. As shown in Table 8, the cores were divided into groups, based on the coring date. We highlight that Group 2 included cores taken from all subsections, while Subsection C was cored four times, from 23 to 796 days after construction.

**Figure 7:**
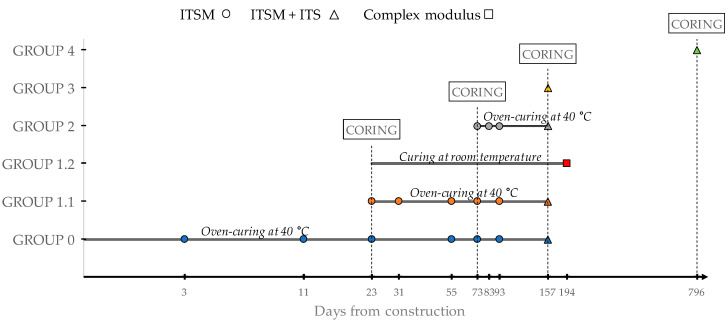
Experimental program.

The gyratory specimens (Group 0) and the core specimens of Groups 1.1 and 2 were oven-cured in the laboratory at 40 °C and tested at different curing periods (Figure 7) for monitoring the evolution of the ITSM. At the end of the selected curing period, the ITS was measured on all specimens. After testing, the volumetric characterisation of Group 0 and Group 2 specimens was performed 157 days after construction, when most of (or all) the hydration had occurred, according to EN 12697-6 [52] (Procedure C, i.e., sealed specimen method) and EN 12697-8 [56].

The three cores of Group 1.2 were cured at room temperature and subjected to complex modulus testing. The cores of Groups 3 and 4 were tested (ITSM and ITS) immediately after coring (Figure 7).

We highlight that the humidity of the CRAM subjected to oven-curing and free evaporation (in laboratory) was totally different from the field, where evaporation is restricted as the mixture was sealed between two bituminous layers (prime coat and tack coat/surface dressing).

### 2.4. Testing Procedures

The ITSM test was carried out at 20 °C, according to EN 12697-26 (Annex C) [57]. The ITSM was measured as average value after the application of five load pulses with a rise time of 124 ms. Two orthogonal diameters were tested for each cylindrical specimen and the average ITSM was calculated.

The ITS test was carried out at 20 °C, according to EN 12697-23 [53]. The test allows the calculation of the tensile strength of a cylindrical specimen, loaded diametrically until its failure.

**Figure 8:**
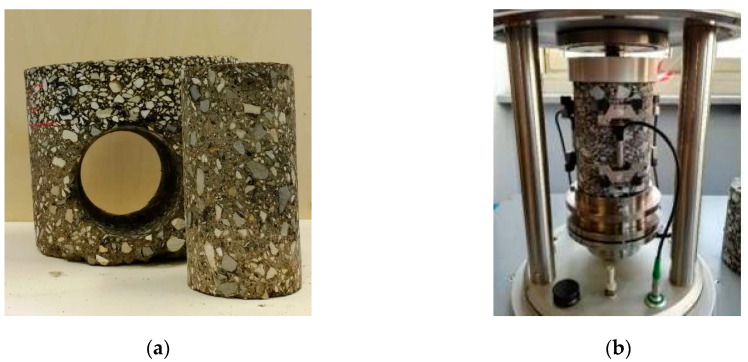
(**a**) Horizontal coring from core of 200-mm diameter; (**b**) detail of the specimen mounted in the asphalt mixtures performance tester (AMPT Pro).

The complex modulus was measured on cylindric specimens with a diameter of 75 mm and a height of 140 mm, obtained with an additional horizontal coring from cores of Group 1.2 (Figure 8a). The test was carried out with the asphalt mixtures performance tester (AMPT Pro). During the test, a strain amplitude of 30 microstrain was kept constant by applying a haversine compression load. Three linear variable differential transformers (LVDT) placed 120° apart (Figure 8b) were used for measuring the axial strain on the middle part of the specimen, while a load cell was employed for axial stress measurement. Five temperatures (0, 10, 20, 35, 50 °C), five frequencies (0.1, 0.5, 1, 5, 10 Hz) and 20 loading cycles for each frequency were applied, allowing the determination of the stiffness modulus *E*_0_ and phase angle *φ*.

## 3. Results and Discussion

### 3.1. Volumetric Characterisation

The bitumen content, recovered from the loose CRAM binder material after in-plant production, was equal to 5.9% with respect to the mixture mass. The air voids content of the CRAM specimens included in Group 0 and Group 2 is shown in Table 9 (average value of 2 or 3 replicates). The air voids of Subsections A, B and C are similar (around 15.5%) and do not allow different site compaction practices to be distinguished. Subsection D, where the least number of passes was carried out (Table 7), provided the highest air voids content (17.0%). On the contrary, Subsection E, where only the 22-ton steel roller was used, provided the lowest air voids content (14.1%).

Apparently, there is a correlation between the air voids of the cores and the number of roller passes. However, we underline that other factors may have influenced the volumetric properties of the specimens, such as the subgrade bearing capacity (existing base course or new CRAM base course) and the post-compaction due to heavy vehicle circulation on the various subsections.

Gyratory specimens provided significantly lower air voids content (11.8%) than core specimens. This suggests that the adopted compaction energy (100 gyrations), which was required by the construction specification [51], overestimates the actual field compaction effort provided by the rollers.

### 3.2. Mechanical Characterisation

#### 3.2.1. ITSM Test Results

**Figure 9:**
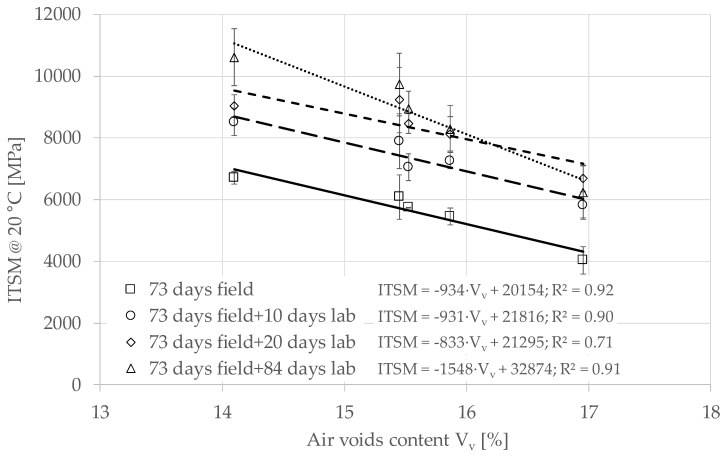
Relationship between indirect tensile stiffness modulus (ITSM) and air voids content of specimens of Group 2 at different curing times.

Figure 9 shows the relationship between ITSM at 20 °C and air voids content of cores of Group 2, at different curing conditions. Each point is the average value of three specimens (Table 8).

The ITSM can be considered linearly dependent on the air voids in all the curing conditions. Results from tests until 93 days after construction (73 days field + 20 days lab), indicate that the curing did not affect the linear correlation between ITSM and air voids. Specifically, a 1% increase in air voids led to a reduction of ITSM of about 900 MPa. Results from tests after a 157-day curing time (73 days field + 84 days lab) kept an almost linear trend but with a higher sensibility to the air voids. This is probably due to the replication of the ITSM tests on the same cores, which may have reduced the stiffness of the cores characterised by a higher air voids (Subsection D with 17% of air voids content).

**Figure 10:**
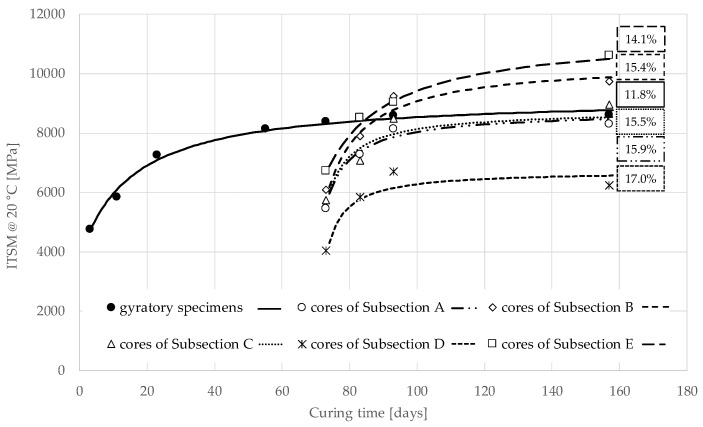
Measured data and model of the evolution of ITSM over curing time for specimens of Group 0 (gyratory) and Group 2 (cores).

Figure 10 shows the evolution of the average ITSM values over the curing time for gyratory specimens (Group 0) and core specimens of Group 2. The experimental data were fitted through the asymptotic model proposed by Graziani et al. [23,58]:

(1) $$y\left(t\right)=y_{i}+\left(y_{a}-y_{i}\right)\frac{t-t_{i}}{(h_{y}-t_{i})\;+\left(t-t_{i}\right)}$$

where *t* (days) is the curing time, *y*(*t*) is the property under investigation (ITSM), *t_i_* is the curing time when the property was measured for the first time (i.e., 3 or 73 days), *y_i_* is an intercept term that represents the value of the properties at the time *t_i_*, *y_a_* is the long-term asymptotic value of *y*(*t*) and *h_y_* (days) is a parameter representing a specific curing time. The parameter *y_i_* gives information on the average rate of evolution of the material properties from the day of construction to *t_i_*.

The agreement between the measured data and the estimated regression curves is quite good, as shown in Figure 10 and Table 10, where the estimated values of the regression parameters are reported along with the coefficient of determination R^2^ and the air voids.

The gyratory specimens of Group 0 and the cores of Group 2 showed a different evolution of ITSM. The gyratory specimens, oven-cured in laboratory at 40 °C starting immediately after production, showed a rapid increase in stiffness in the first days, with the ITSM reaching about 4500 MPa after 3 days and 7200 MPa after 21 days. At longer curing times, the stiffness increase slowed down, with the ITSM reaching about 8200 MPa after 73 days and a long-term asymptotic value of 9246 MPa (Table 10).

For the cores, we have the first ITSM measurement after 73 days of field curing, with values ranging from about 4000 (Subsection D) to 6500 MPa (Subsection E). At this curing time, the difference in ITSM between cores and gyratory specimens, is due to differences in both volumetric properties (the cores had higher voids) and curing conditions (oven-curing vs. field curing). The effect of air voids can be estimated using the experimental data reported in Figure 9. Specifically, with an air voids content of 11.8% we can extrapolate an ITSM of about 9100 MPa after 73 days of field curing. Such value is even higher than the ITSM measured on the gyratory specimens. We can conclude that, in these in-situ conditions (pavement stratigraphy, location and climate), field curing did not penalise the stiffness evolution until 73 days. This tendency is confirmed in the long-term. In fact, Figure 10 shows that the core specimens, which were oven-cured at 40 °C after extraction, had a rapid increase in stiffness and reached an asymptotic value of ITSM ranging from 6729 (Subsection D) to 11,375 MPa (Subsection E). We observe that the asymptotic value of the gyratory specimens is comprised in this range, although they had lower air voids.

**Figure 11:**
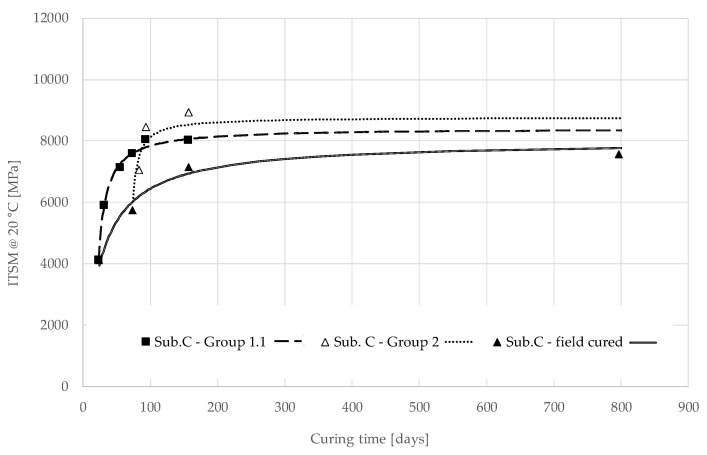
Measured data and model of the evolution of ITSM over curing time for core specimens of Subsection C: Group 1.1, Group 2 and field curing.

The field curing curve shown in Figure 11 depicts the ITSM evolution observed on core specimens taken from Subsection C, from 23 to 796 days after construction, when also field ageing, moisture damage and traffic induced stress influenced the material performance. These measurements reveal the actual stiffness evolution of the material in the field. This trend is compared with the stiffness evolution observed on cores taken 23 days (Group 1.1) and 73 days (Group 2 Sub. C) after construction and then oven-cured at 40 °C. The measurements were fitted using the model described in Equation (1) and the estimated values of the regression parameters are reported in Table 10. The core specimens taken after 23 and 73 days and oven-cured at 40 °C show a rapid increase in stiffness and, in the long term, they are characterised by similar asymptotic values of ITSM: 8415 MPa and 8775 MPa, respectively. These values are only slightly higher than the asymptotic value which characterises the field cured material, 8001 MPa.

In summary, oven-curing with free evaporation and high temperature (40 °C), a procedure that is often used for accelerated curing in the laboratory, may lead to an underestimation of the long-term stiffness of the mixture cured in the field with restricted evaporation (Figure 10). On the other hand, if oven-curing is applied after a few days or weeks of field curing with restricted evaporation, the long-term stiffness will not be affected (Figure 11). This is because the humidity of the material subjected to oven-curing and free evaporation may be totally different from the field. Therefore, the evolution of the cementitious and bituminous bonds will be different because humidity enhances the former and penalises the latter.

These results suggest that the curing conditions immediately after compaction determine the microstructure of the material (distribution and location of cementitious and bituminous bonds). With immediate oven-curing and free evaporation (laboratory conditions), bituminous bonds are favoured with respect to cementitious bonds, whereas if evaporation in restricted (field curing), the cementitious bonds are favoured leading to a higher long-term stiffness. When oven-curing is applied a few days/weeks after compaction (i.e., when the mixture microstructure is already formed in sealed conditions), the stiffness development will be accelerated with a small effect on its long-term value.

#### 3.2.2. ITS Results

**Figure 12:**
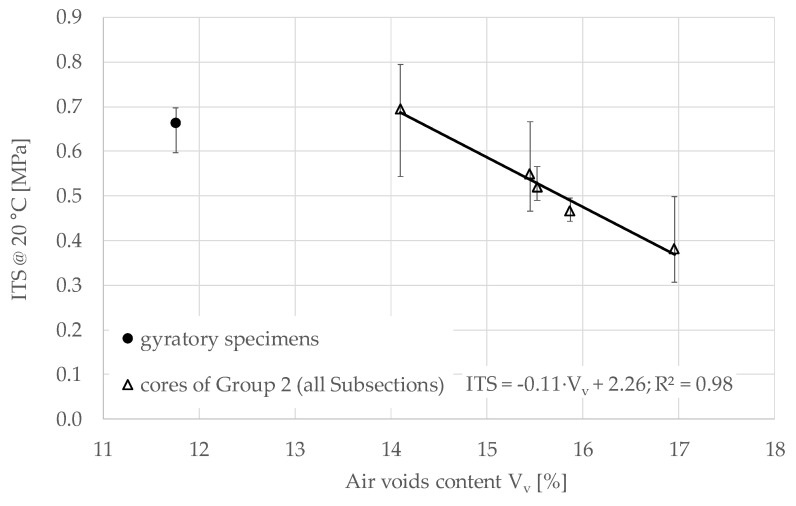
Indirect tensile strength (ITS) (with max and min error bars) as a function of air voids, 157 days after construction.

Figure 12 shows the results of the ITS tests performed on the core specimens of Group 2, cured 73 days in the field and then 84 days in laboratory at 40 °C, and on the gyratory specimens cured 157 days in laboratory at 40 °C. A linear relationship is found between the ITS of the cores and their air voids content. Specifically, a 3% increase in air voids led to approximately a halving of ITS (from 0.69 to 0.38 MPa). This trend shows that if hypothetical cores with an air void content of 11.8% (corresponding to the value obtained for the gyratory specimens) were considered, the ITS value would be considerably higher than that provided by the gyratory specimens. This confirms what was already observed during ITSM tests: the combination of field and laboratory curing would lead to a different structure compared to the sole curing in the laboratory at 40 °C.

#### 3.2.3. Complex Modulus

**Figure 13:**
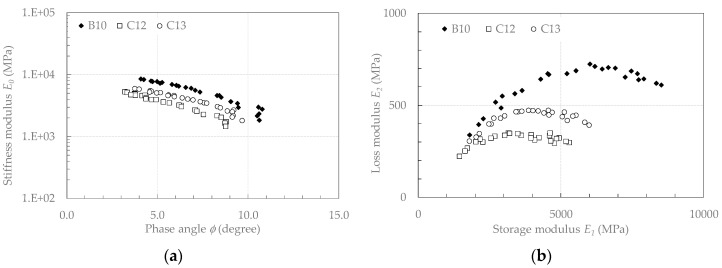
Measured values of E_0_ for CRAM: (**a**) Black diagram; (**b**) Cole–Cole diagram

Figure 13 shows the complex modulus results obtained on cores extracted from Subsections B (B10, with air voids content of 14.3%) and C (C12 and C13, with air voids contents of 18.4% and 18.9%, respectively). The results are plotted in the Black diagram (stiffness modulus $E_{0}\;$ as a function of the phase angle ϕ–Figure 13a) and in the Cole–Cole diagram (loss modulus $E_{2}$ as a function of storage modulus $E_{1}$–Figure 13b).

Overall, the stiffness modulus was comprised between 9500 (B10, *T* = 0 °C, *f* = 10 Hz), and 1500 MPa (C12, *T* = 50 °C, *f* = 0.1 Hz). This range of variation is less than one order of magnitude, whereas, in the same temperature and frequency range, conventional AC mixtures normally show a variability of more than two orders of magnitude [59]. Concurrently, the values of the phase angle were less than about 10°. These reduced ranges of variability characterise the typical behaviour of cold recycled mixtures where cement plays an important role in limiting the thermo-viscoelastic behaviour [60].

For each specimen, a loading frequency increase (or decrease) had the same effect on $E^{*}$ as the temperature decrease (or increase). This confirms the validity of the time-temperature superposition principle (TTSP). In other terms, despite the concurrent presence of the residual bitumen from the emulsion and the aged bitumen of the RA aggregates, the tested CRAMs showed a thermo-rheologically simple behaviour. The master curves of the stiffness modulus and phase angle were obtained by fixing the reference temperature *T*_0_ = 20 °C and shifting the experimental data along the frequency axis until obtaining continuous curves. For each testing temperature *T* the amount of shifting, i.e., the shift factors $a_{0}\left(T\right)$, was obtained through the closed form shifting (CFS) algorithm, consisting in the minimisation of the area between two adjacent isothermal curves of *E*_0_ [61]. The same shift factors were used also for shifting the *φ* values.

**Figure 14:**
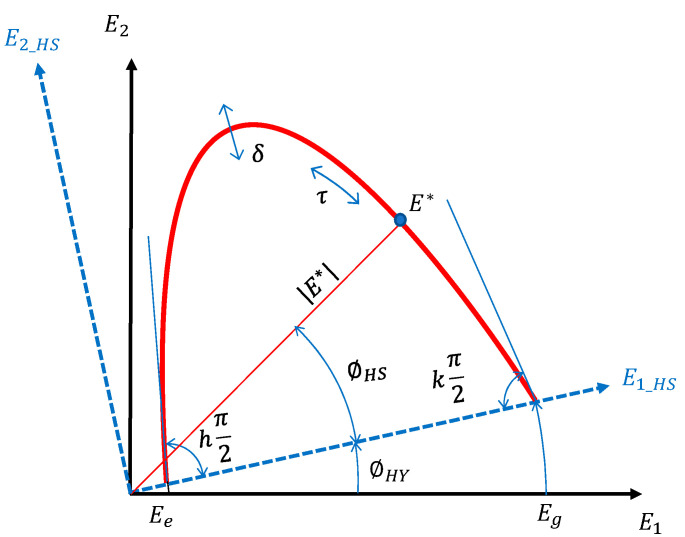
Comparison between the Huet–Sayegh (HS) and HS-HY model.

Rheological modelling was carried out using the model proposed by Graziani et al. [10,11] that combines viscoelastic and hysteretic dissipation mechanisms. The viscoelastic part is represented by the Huet–Sayegh (HS) model [62] while the hysteretic part (HY) is represented by a time- and temperature-independent phase angle ($\phi_{HY}$). The model, abbreviated HS-HY, is described by the following equation:

(2) $$E_{HS-HY}^{*}\left(\omega \right)=\left[E_{e}+\frac{E_{g}-E_{e}}{1+\delta {\left(j\omega \tau \right)}^{-k}+{\left(j\omega \tau \right)}^{-h}}\right]\cdot \exp\left(j\phi_{HY}\right)$$

where the term in square brackets describes the HS model, and the term $\exp\left(j\phi_{HY}\right)$ represents a rotation in the complex plane (Figure 14). Physically, the phase angle $\phi_{HY}$ accounts for the time-independent (non-viscous) and temperature-independent dissipation phenomena which are present during cyclic loading. The term hysteretic is normally used to indicate this type of dissipation mechanism that may be attributed to the cementitious bonds or to internal friction phenomena [63,64].

In Equation (2), j is the imaginary unit and ω is the angular frequency (ω=2πf). *E_e_* is the equilibrium value of $E^{*}$ (ωτ → 0) and represents the purely elastic material response when the bitumen is liquid, and its contribution to stiffness vanishes (high temperature/low frequency). For AC mixtures this value represents the interlock between aggregates [65] and for CRAMs it also accounts for the effect of cementitious bonds. *E_g_* is the glassy value of $E^{*}$ (ωτ → ∞) and represents the purely elastic material response when the bitumen is a glassy solid (low temperature/high frequency). Its value is mainly affected by the volumetric properties of the mixture [66].

The dimensionless parameters *k, δ* and *h* control the shape of the model in the low, intermediate and high temperature range [67]. Higher values of *h* and *k* indicate that a higher viscous dissipation component is present in the material response (with one indicating purely viscous behaviour). On the other hand, lower values of *h* and *k* indicate that the material response is more elastic (with zero indicating purely elastic behaviour). In general, physically consistent values are 0 < *k* < *h* < 1, with *k* characterising the low temperature/high frequency behaviour and *h* the high temperature/low frequency behaviour.

The characteristic time τ is a function of the testing temperature:

(3) $$\tau =\tau_{0}\cdot a_{0}\left(T\right)$$

where $\tau_{0}$ is the characteristic time at the reference temperature. The value of τ does not affect the shape of the master curves. In fact, since τ is a frequency multiplier, it only affects the position of the master curve along the frequency axes. If τ increases, the *E*_0_ master curve shifts to the left indicating a lower relaxation ability of the material. Higher values of τ have been correlated to higher degree of bitumen aging and higher RA content, in both hot and cold mixtures [10,68,69,70].

**Figure 15:**
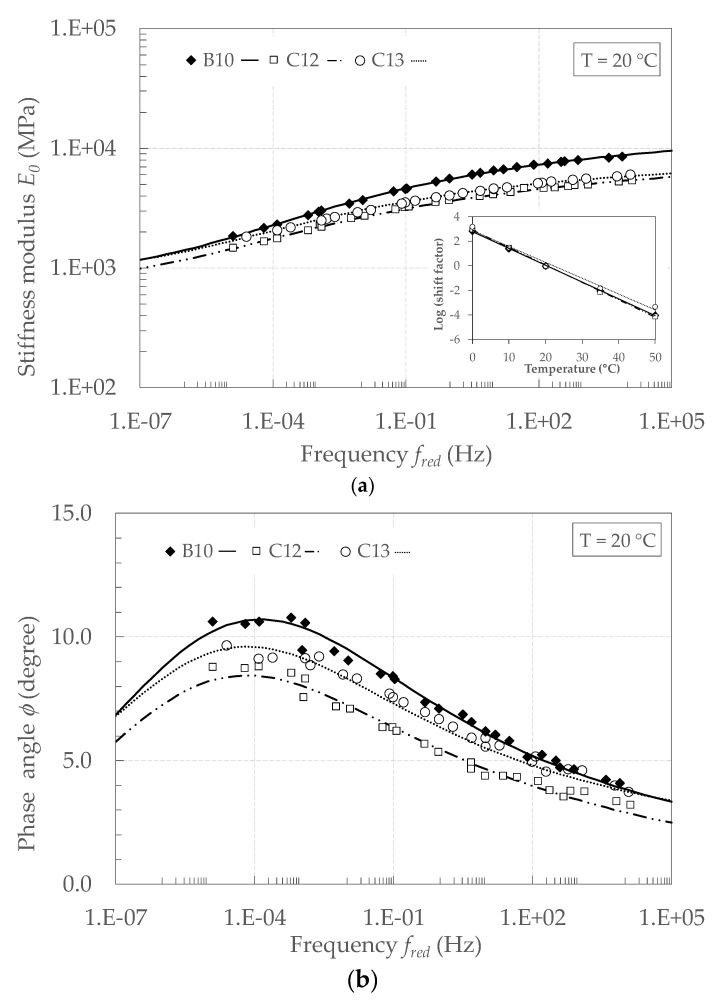
Master curves of CRAM at 20 °C: (**a**) stiffness modulus *E_0_*; (**b**) phase angle *ϕ*.

Figure 15a shows the master curves superimposed to the experimental data and the shift factors, whereas Table 11 summarises the model parameters. The *E_e_* values of the three specimens are very similar, confirming that they were obtained from the same mixture (same aggregate composition, same amount of cement). Very similar *E_g_* values were obtained for C12 e C13 specimens whereas B10 specimen provided a higher value, probably due to a lower air voids content of B10 (14.3%) with respect to C12 and C13 (18.4 and 18.9%, respectively).

Identical values of *h* and *k* were used for all specimens, obtaining an excellent fitting of the experimental data. This indicates that when ωτ → 0 (high temperature/low frequency) and ωτ → ∞ (low temperature/high frequency) the specimens showed the same viscoelastic dissipation behaviour. The variability of $\tau_{0}$ may be explained by specimen-to-specimen variability.

The values of $\phi_{HY}$ are comprised between 0.7° and 1.5°, whereas the measured phase angle ranges from 3° to 11° (Figure 15b). Its means that the ratio $\phi_{HY}/\phi$ varies from less than 10% (high temperature/low frequency) to about 30% (high temperature/low frequency). We may conclude that the hysteretic dissipation component represents an important part of the material dissipation behaviour and, more in general, the material is characterised by an intermediate behaviour between AC mixtures and cement-bound mixtures.

## 4. Conclusions

This study aimed at comparing the oven-curing in laboratory and the field curing of a CRAM mixture, laid in a full-scale trial section. The CRAM was produced with the CCPR technique and applied as binder course. Gyratory compacted specimens and cores taken from the pavement at different time intervals were investigated for more than two years.

The results show that the air voids content strongly affects the mechanical properties of CRAMs. Linear relationships link both ITSM and ITS to the air voids content, by showing that the production of laboratory specimen with a correct air voids level is essential for estimating the mixture performance in the field.

The evolution of the average ITSM values over the curing time was fitted with an asymptotic model, which was in good agreement with the measured data. The differences in curing procedures between laboratory and field caused differences in ITSM. This suggests that oven-curing with free evaporation (laboratory) and sealed curing with restricted evaporation (field) led to the formation of a different microstructure. Thus, when bitumen emulsion and cement are used as co-binders, laboratory curing should be carried out both in sealed and unsealed condition to have a complete understanding of the mixture potential behaviour in the field.

The complex modulus results confirmed the validity of the time-temperature superposition principle. A rheological model, explicitly considering non-viscous dissipation, was adopted for analysing the data and confirmed that CRAMs are characterised by an intermediate behaviour between AC and cement-bound mixtures.

This study showed that an additional effort is necessary to find a more effective procedure for producing CRAM specimens in laboratory, able to appropriately estimate the field material performance.

## Figures and Tables

**Table 1:** Reclaimed asphalt (RA) aggregate characteristics.

Parameter	Standard	Value
Aggregate size (mm)	EN 933-1 [28]	16
Passing at 0.063 mm (%)	EN 933-1 [28]	5.6
Flakiness index (%)	EN 933-3 [29]	7.3
Shape index (%)	EN 933-4 [30]	5.7
Crushed aggregate particle (%)	EN 933-5 [31]	100
Sand Equivalent (%)	EN 933-8 [32]	70.6
Resistance to fragmentation (%)	EN 1097-2 [33]	17
Water absorption (%)	EN 1097-6 [34]	1.3
Bitumen content to mixture (%)	EN 12697-1 [35]	4.1

**Table 2:** Fine aggregate characteristics.

Parameter	Standard	Value
Fines content (%)	EN 933-1 [28]	2.0
Sand equivalent (%)	EN 933-8 [32]	81
Methylene blue (g/kg)	EN 933-9 [36]	1.2
Density (Saturated surface dry) (Mg/m^3^)	EN 1097-6 [34]	2.64
Loose bulk density (Mg/m^3^)	EN 1097-3 [37]	1.56
Water absorption (%)	EN 1097-6 [34]	1.0

**Table 3:** Filler characteristics.

Parameter	Standard	Value
Passing at 2 mm (%)	EN 933-1 [28]	100
Passing at 0.125 mm (%)	EN 933-1 [28]	98
Passing at 0.063 mm (%)	EN 933-1 [28]	95
Methylene blue (g/kg)	EN 933-9 [36]	3.5
Fineness (Blaine) (cm^2^/g)	EN 196-6 [38]	6500

**Table 4:** Portland cement characteristics.

Parameter	Standard	Value	Specifications
Setting time (min)	EN 196-3 [41]	140	>75
Fineness (Blaine) (cm^2^/g)	EN 196-6 [38]	4900	-
Strength after 2 days (MPa)	EN 196-1 [42]	22	>10
Strength after 28 days (MPa)	EN 196-1 [42]	42	≥32.5

**Table 5:** Bituminous emulsion characteristics.

Parameter	Standard	Value	Specification
pH value (pH)	EN 12850 [43]	2.45	positive
Residual binder (%)	EN 1431 [44]	59.8	60 ± 2
Storage stability (%)	EN 1429 [45]	3	≤10
Breaking value (–)	EN 13075-1 [46]	190	>150
Mixing stability with cement (g)	EN 12848 [47]	<0.2	<2
**Characteristic of bitumen (EN 13074 [48])**			
Penetration value (mm × 10^−1^)	EN 1426 [49]	58	<100
Softening point (°C)	EN 1427 [50]	47.8	>45

**Table 6:** Design gradation and specification limits.

Sieve Size (mm)	Design Gradation (% Passing)	Specification Limits (% Passing)
31.5	100	100–100
20	99	90–100
16	96	
14	93	
12.5	88	
10	79	50–80
8	71	
6.3	62	
5	54	
4	46	30–55
2	33	20–40
0.5	19	10–25
0.25	16	
0.063	5.1	3–8

**Table 7:** Compaction procedure of the cold recycled asphalt mixture (CRAM) binder course.

Subsection	First Phase		Second Phase		Third Phase	
	Rolle Type	# Passes	Rolle Type	# Passes	Rolle Type	# Passes
A	9t SR ^1^	5	22t PR ^2^	10	9t SR	5
B	9t SR	5	22t PR	15	9t SR	5
C	22t PR	2	9t SR	5	22t PR	13
D	9t SR	3	22t PR	15		
E	12t SR ^3^	22				

^1^ Nine-ton steel-wheeled roller; ^2^ 22-ton pneumatic-tired roller; ^3^ 12-ton steel-wheeled roller.

**Table 8:** Coring plan.

Group	Coring Date (mm/dd/yy)	Days from Construction	Core Diameter (mm)	Number of Specimens				
				Subsections				
				A	B	C	D	E
1.1	16 April 2018	23	150	-	-	3	-	-
1.2	16 April 2018	23	200	-	1	2	-	-
2	5 June 2018	73	134	3	3	3	3	3
3	28 August 2018	157	134	-	3	3	-	-
4	28 May 2018	796	134	-	-	3	-	-

**Table 9:** Air voids content of CRAM binder specimens of Group 0 and Group 2.

Specimens	Air Voids Content	
	Average Value (%)	Max-Min (%)
Gyratory	11.8	0.853
Subsection A	15.9	0.789
Subsection B	15.4	1.278
Subsection C	15.5	0.642
Subsection D	17.0	0.561
Subsection E	14.1	1.008

**Table 10:** Estimated values of the regression parameters and air voids content of specimens of Group 0, Group 1.1, Group 2 and field curing.

Specimen Type	*t_i_*	*y_i_*	*y_a_*	*h_y_*	*R* ^2^	Air Voids Content
	(Days)	(MPa)	(MPa)	(Days)	(-)	(%)
gyratory specimens	3	4668	9246	21	0.97	11.8
cores of Sub. A-Group 2	73	5603	8730	81	0.85	15.9
cores of Sub. B-Group 2	73	5807	10,412	84	0.75	15.4
cores of Sub. C-Group 2	73	5625	8775	80	0.85	15.5
cores of Sub. D-Group 2	73	3815	6729	78	0.70	17.0
cores of Sub. E-Group 2	73	6643	11,375	92	0.89	14.1
cores of Sub. C-Group 1.1	23	4139	8415	35	0.94	-
cores of Sub. C-field curing	23	3850	8001	69	0.94	-

**Table 11:** Huet–Sayegh model parameters (T_ref_ = 20 °C).

Specimen	E_g_	E_e_	k	h	δ	log τ_0_	$\phi_{HY}$
	(MPa)	(MPa)	-	-	-	-	(°)
B10	13,583	833	0.093	0.265	1.819	0.669	0.701
C12	7921	712	0.093	0.265	1.990	1.420	0.147
C13	7943	861	0.093	0.265	1.600	1.566	1.439

## Acronyms

The following acronyms were used in the text

**Definition**	**Acronym**
closed form shifting algorithm	CFS
cold central-plant recycling	CCPR
cold recycled asphalt mixture	CRAM
cold recycled material	CRM
cold in-place recycling	CIR
full-depth reclamation	FDR
Huet–Sayegh model	HS
indirect tensile stiffness modulus	ITSM
indirect tensile strength	ITS
linear variable differential transformers	LVDT
